# Supplementary material for: Mechanical Properties, Fractal Dimension, and Texture Analysis of Selected 3D-Printed Resins Used in Dentistry That Underwent the Compression Test
Source: Polymers (Basel). 2023 Apr 2;15(7):1772. doi: 10.3390/polym15071772 (PMC10098904; doi:10.3390/polym15071772)
Supplement: Supplementary file 1 [file polymers-15-01772-s001.zip › polymers-2252345-supplementary.pdf]

7. Supplementary

Table S1. A brief description of the characteristics and applications of selected 3D printing resins in dentistry, as suggested by the manufacturer.

Resin	Details
**Dental LT Clear Resin**	Characteristics: long-term, biocompatible, transparent, not suitable for sterilization, highly esthetic, rigid<br><br>Use: hard splints, occlusal guards, direct-printed orthodontic appliances—aligners, retainers
**BioMed Amber Resin**	Characteristics: biocompatible for short-term skin or mucosal<br><br>contact, yellowish and transparent<br><br>Use: strong, functioning threads, end-use medical devices, specimen collection kits, surgical guides, implant sizing models
**IBT Resin**	Characteristics: biocompatible, for short-term contact, elastic, flexible, transparent, translucent<br><br>Use: indirect bonding trays, rapid dental bracket placement, high-quality orthodontics, tooth templates, guides

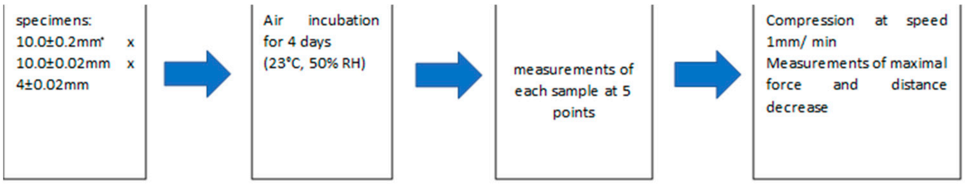

Figure S1. Axial compression test performed on the presented materials, according to the PN-EN ISO 604:2003 standard (*F*, force (N); *A*, initial

cross-sectional area measurement (mm^2); L, measurement of the distance between compression plates (mm); ΔL, decrease in distance between the plates (mm)).

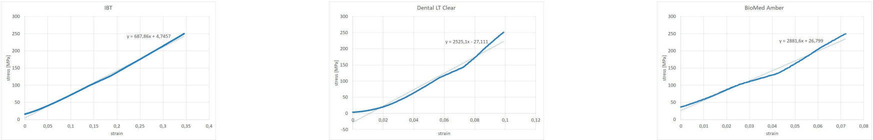

Figure S2. Nominal strain diagram of the three examined materials.

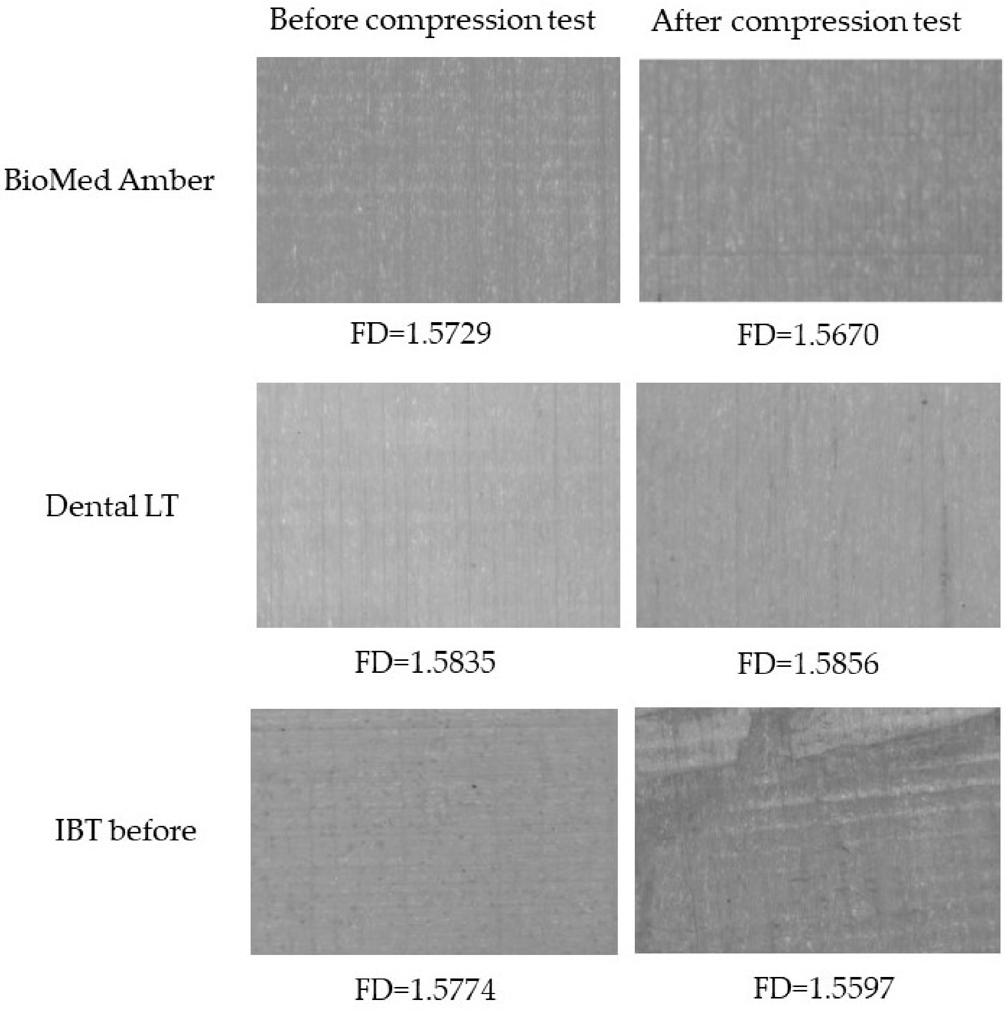

Figure S3. Microscopic pictures of the examined materials and the fractal dimension value of each surface after and before compression testing.

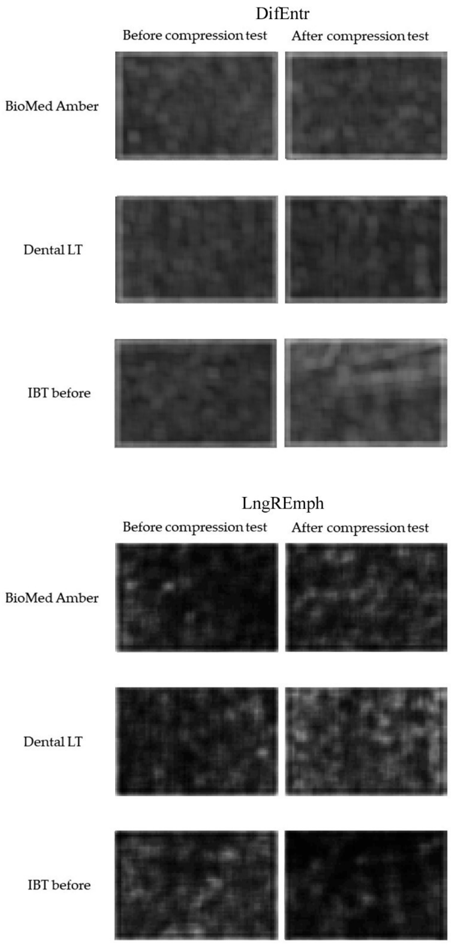

Figure S4. Texture maps obtained after analyzing images of the surfaces of the tested materials. The lighter areas indicate a stronger local expression of the studied image structure feature, while the darker areas show a low local level of feature.
